# Regulation of multi-organ inflammation in the regulatory T cell-deficient scurfy mice

**DOI:** 10.1186/1423-0127-16-20

**Published:** 2009-02-12

**Authors:** Rahul Sharma, Sun-sang Joe Sung, Shu Man Fu, Shyr-Te Ju

**Affiliations:** 1 Center for Immunity, Inflammation, and Regenerative Medicine, and Department of Medicine, University of Virginia, Charlottesville, VA 22908, USA

## Abstract

Scurfy mice display the most severe form of multi-organ inflammation due to total lack of the CD4^+^Foxp3^+ ^regulatory T cells (Treg) resulted from a mutation of the X-linked transcription factor Foxp3. A large repertoire of Treg-suppressible, inflammation-inducing T cells was demonstrated by adoptive transfer experiments using *Rag1*^-/- ^mice as recipients and by prolongation of lifespan through breeding with *Fas*^*lpr*/*lpr *^mutant. Inflammation in the ear, eyes, skin, tail, salivary glands, lungs, stomach, pancreas, liver, small intestine, colon, skeletal muscle, and accessory reproductive organs are identified. Genetic and cellular regulations of specific organ inflammation are described. Sf mice may be useful for the identification of organ-specific antigens and Treg capable of suppressing inflammation in an organ-specific manner. Sf mice are also useful to determine the important inflammation process at the checkpoint after Treg regulation using genetic analysis through breeding.

## Review

Thymic selection imparts T cell subsets with distinct functions and specific antigenic markers. In the thymus, the CD4^+ ^T cell compartment contains a Foxp3^- ^population (termed conventional T cells or Tconv) that when exits to the periphery will respond to antigenic stimulation and a Foxp3^+ ^population (regulatory T cells or Treg) that when exits to the periphery will suppress immune responses [reviewed in [1-3]]. Although negative selection eliminates the high-affinity T cells that react with self antigens, T cells reactive with self antigens with low to moderate affinity may escape negative selection and exit to the periphery where they may become auto-reactive under appropriate conditions [4,5]. The presence of Treg is necessary to suppress this response to maintain peripheral tolerance. Early works using day-3 thymectomy or anti-CD25 antibody treatment have implicated Treg presence [6,7], but the most convincing and definitive evidence for Treg came from the genetic studies of Scurfy (Sf) mice and IPEX patients, both display fatal multi-organ inflammation caused by mutations in the transcription factor Foxp3 [8,9]. Foxp3 is absolutely required for the Treg generation in the thymus and Treg maintenance in the periphery. In this regard, the demonstration of inducible Treg from naïve CD4^+^CD25^-^Foxp3^- ^T cells in the periphery implies that Sf mice and IPEX patients must also lack inducible Treg [10]. Inducible Treg may be more important for the regulation of immune response to foreign antigens or foreign antigens steadily associated with the host. Thus, Foxp3 expression is required lifelong for the maintenance of tolerance [11]. Foxp3 expression defect may result in spontaneous response to both self antigens and foreign antigens steadily represented in the host.

The overall Treg expression in the periphery is affected by factors that participate in the Treg generation in the thymus and by factors that maintain Treg expression in the periphery. In addition to Foxp3, thymic Treg generation depends on high-affinity IL-2 signaling [12]. In the absence of IL-2 or IL-2Rα, thymic Treg generation is reduced but IL-7 signaling can partially compensate this defect [12]. Another study using Foxp3-GFP knock-in mice also favors the interpretation that IL-2 is not indispensable for the thymic Foxp3^+ ^Treg generation [13]. Naïve CD4^+^Foxp3^- ^T cells in the periphery could be converted to functional CD4^+^Foxp3^+ ^T cells when activated through TCR and in the presence of IL-2 and TGF-β1 [10]. Peripheral Treg are maintained to a great extent by the high affinity IL-2 and TGF-β1 signaling pathways [14-16]. In the periphery, IL-2 defect can be compensated to a significant extent by the IL-15/IL-15R signaling pathway but the requirement of TGF-β1 is indispensable [10,12,17]. Many factors that regulate Treg development and homeostasis also regulate Tconv cells. Because of this complicated etiology and regulation, Treg expression in different mutant strains varied and its effect on peripheral tolerance results in varying manifestation of multi-organ inflammation. We will focus on the multi-organ inflammation associated with *Il2*^-/- ^and Sf mice in the B6 genetic background because they have been extensively studied.

Spontaneous multi-organ inflammation is a characteristic of Treg deficiency and Sf mice provide the best source to study such process. A contentious issue is whether the multi-organ inflammation responses are the results of activation of organ-specific auto-reactive T cells. Critically speaking, these spontaneous inflammation responses cannot be considered autoimmune without the identification of the target antigens and their organ-specific association. Perhaps the best example that Treg control auto-immune response is the gastritis induced by day-3-thymectomy that activates both T and B cell responses against the H^+^/K^+^-ATPase of stomach parietal cells [18,19]. In experimental autoimmune prostatitis and oocytitis, specific responses against EAPA and MATER organ antigens have been implicated [20,21]. Antibodies reactive against a mitochondrial antigen associated with cholangitis have been demonstrated in Sf mice [22]. Because Treg controls immune responses to both self and foreign antigens, it is likely that the multi-organ inflammation is the result of loss of tolerance to self antigens as well as foreign antigens that are more often associated with particular organs in the host including the steady presence of antigens from the environment, food, bedding, and microbiota [23].

Sf mice display the most severe form of spontaneous multi-organ inflammation disease but their multi-organ inflammation is restricted to a few. Sf mice die around 24–28 days old with severe inflammation in the ear, conjunctiva, skin, lungs, liver and tail. [24]. Common autoimmune diseases such as thyroiditis, diabetes, encephalomyelitis, arthritis, glomerulonephritis and inflammation in the oral and gastrointestinal tracts are not observed [24,25]. Two approaches were used to determine if early death or pre-weaning condition prevented the expression of inflammation in these organs. The lifespan of Sf mice can be prolonged to 8–20 weeks old by introducing the apoptosis-preventing *Fas*^*lpr*/*lpr *^gene [26]. Surprisingly, additional inflammation was observed only in colon and accessory reproductive organs. Thus, pre-weaning condition may not be the only factor that affects organ-specific expression of inflammation. The second method is by transferring Sf lymph node cells into *Rag1*^-/- ^recipients [25]. This approach not only induced severe inflammation in the skin, lung and liver but also in salivary gland, stomach, pancreas, small intestine, and colon. Thus, Sf mice probably contain many inflammation-inducing T cells capable of inducing inflammation in various organs. Some of the organs that are not inflamed are probably caused by factors other than early death or the absence of inflammation-inducing T cells [25].

**Figure 1:**
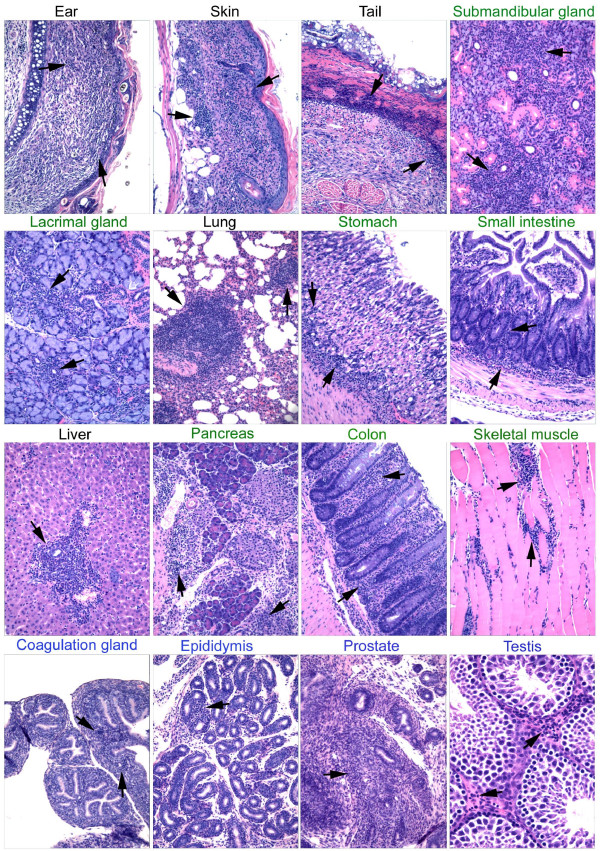
**Multi-organ inflammation in Sf mice, Sf.*Fas*^*lpr*/*lpr *^mice, and *Rag1*^-/- ^recipients of Sf lymph node cells**. Each panel represents an inflamed target organ. Target organs were indicated by color: Black indicates spontaneous inflammation in Sf mice. Green indicates organs of *Rag1*^-/- ^recipients of intravenous adoptive transfer (Pancreas is obtained from a recipient of intraperitoneal transfer). Blue indicates accessory reproductive organs from 10 weeks old Sf.*Fas*^*lprlpr *^male that lived beyond weaning. Arrows indicate areas of leukocyte infiltration. Magnification is 10× for all except for testis which is 20×.

For simplicity and clarity purpose, a summary figure (Figure 1) is used to present the inflammation of various organs induced under various conditions. In each presentation, leukocyte infiltration is indicated by arrows. Negative controls totally lack leukocyte infiltration and they are not presented. Description of each panel is included in the figure legend.

### Skin inflammation

Skin inflammation is the earliest external symptom observed in Sf mice. The severely inflamed areas are ear, eyelids, and tail. Conjunctivitis is probably caused by frequent scratching which also worsened inflammation in areas surrounding the ears and eyes. Adoptive transfer of Sf lymph node cells into adult *Rag1*^-/- ^recipients induced skin inflammation in which eyelids are the first to appear. In contrast, tail inflammation is minimal if any, suggesting organ development control of tail inflammation. This is supported by the expression of tail inflammation following adoptive transfer of Sf lymph node cells into neonate *Rag1*^-/- ^recipients [25]. Skin inflammation in Sf mice is strongly associated with Th2 type of immune response with high serum IgE [27], but the Th2 type cytokines and IgE expression occurred with simultaneous increase of Th1 type response. Perhaps the most interesting observation is the lack of skin inflammation in Sf.*Il2*^-/- ^mice which are generated by breeding *Il2*^-/- ^gene into the Sf mice. Sf.*Il2*^-/- ^lymph node cells also failed to induce skin inflammation upon transfer into *Rag1*^-/- ^recipients. Thus, IL-2 is required for the induction of skin inflammation in the absence of Treg [26].

### Inflammation in salivary and lacrimal glands

Sjögren's syndrome is characterized by inflammation in the salivary glands and lacrimal glands with dry mouth and dry eyes, respectively. *Il2*^-/- ^and *Il2rα*^-/- ^mice develop inflammation in these glands and their ability to produce saliva upon stimulation with Pilocarpine is impaired [28]. Interestingly, Sf mice do not develop inflammation in these glands but transfer of Sf lymph node cells into *Rag1*^-/- ^recipients induced strong inflammation in the lacrimal glands and salivary glands and inhibited salivation function, suggesting the presence of inflammation-inducing T cells against lacrimal and salivary glands [28]. The submandibular gland (SMG) is the major mouse saliva-producing gland and its development is age-dependent and sexually dimorphic [29]. The acini of the SMG develop soon after birth and dominate in the early phase of SMG development. The granular convoluted tubules (GCT) develop around 3–4 weeks after birth and the expression is markedly stronger in male than in female mice. Infiltration in the salivary glands in *Il2*^-/- ^mice as well as *Rag1*^-/- ^recipients of Sf lymph node cells was observed primarily in the areas of acini but also noticeable around the GCT areas, with destruction and disappearance of the acini and atrophy of GCT [28]. We noticed that the SMG in Sf mice is not only free from inflammation but also growth-arrested in that the male-dominant expression of GCT is inhibited, leaving the organ mostly occupied by the acini. Several observations indicate that GCT development is not important for SMG inflammation. First, treatment of the long-lived Sf.*Fas*^*lpr*/*lpr *^mice with testosterone fully restored GCT development but still failed to induce inflammation in the SMG. Second, SMG inflammation was observed in *Il2*^-/- ^mice that also have a greatly reduced GCT expression (but not as severe as Sf mice). Third, treatment of Sf mice with daily oral application of LPS or Poly:I/C induced SMG inflammation in the absence of GCT development [30]. The latter observations suggest that defect in innate immunity and antigen-presentation may be involved in the inhibition of inflammation in the salivary and lacrimal glands in Sf mice.

### Lung inflammation

Lung inflammation in Sf mice is characterized by the severe infiltration of leukocytes around the bronchiole and alveoli. Both peri-vascular and parenchymal infiltrations were observed. In contrast, lung inflammation was not observed in *Il2*^-/- ^mice and breeding *Il2*^-/- ^into Sf mice (Sf.*Il2*^-/-^) inhibited lung inflammation, a situation reminiscent of skin inflammation [26]. A recent study has demonstrated that lacking IL-10-producing Treg is inductive to skin and colon inflammation [31]. However, a normal level of IL-10 mRNA expression was observed in the Treg of *Il2*^-/- ^mice [13]. Our observation with *Sf.Il2*^-/- ^mice that lack Treg also ruled out the possibility that the residual Treg in *Il2*^-/- ^mice express IL-10 and that lung inflammation is inhibited due to the presence of such IL-10-producing Treg.

In contrast to *Il2*^-/- ^mice, *Il2rα*^-/- ^mice display severe lung inflammation. Although the reason is unknown at present, *Il2rα*^-/- ^differs from *Il2*^-/- ^mice in that they over-express CD8^+ ^memory T cells that occupy more than 75% of the total T cell repertoire [32]. It is tempting to speculate that these CD8^+ ^T cells are responding to air and environmental antigens such as virus that are presented through class-I antigen processing pathway by the lung antigen-presenting cells. Whether the over-expressed CD8^+ ^T cells play a role in lung inflammation remains to be determined.

### Gastritis and small intestine inflammation

Gastritis and small intestine inflammation are neither observed in Sf mice nor in the Sf.*Fas*^*lpr*/*lpr *^mice that have a prolonged lifespan beyond weaning. However, they were induced by transfer of Sf lymph node cells into *Rag1*^-/- ^recipients [25]. Moreover, gastritis and small intestine inflammation were not observed in *Il2*^-/- ^mice and transfer of Sf.*Il2*^-/- ^lymph node cells failed to induce inflammation in the stomach and small intestine. Thus, adoptive transfer of lymph node cells from Sf and Sf.*Fas*^*lpr*/*lpr *^mice into *Rag1*^-/- ^recipients remains the only protocol capable of inducing gastritis and small intestine inflammation. Although we observed inflammation around the areas containing the parietal cells of the stomach, whether this inflammation includes a component against H^+^/K^+ ^ATPase, like the case in the day-3 thymectomized Balb/c mice, is unknown at present.

### Liver inflammation and cholangitis

Both *Il2*^-/- ^and Sf mice develop liver inflammation manifested by peri-vascular infiltration of leukocytes. High titers of all isotypes against anti-mitochondrial protein (pyruvate dehydrogenase complex component E2) characteristically associated with cholangitis were identified in the sera of Sf mice [22]. Leukocytic infiltration was observed around portal area with damage in the biliary duct. Livers from Sf mice strongly expressed inflammatory cytokines including TNF-α, IFN-γ, IL-6, IL-12 and IL-23 [22]. Presence of anti-mitochondrial protein antibodies were also detected in *Il2*^-/- ^and Sf.*Il2*^-/- ^mice but the histology and cytokine analyses of liver samples are not completed. Autoimmune cholangitis was also observed in *Il2Rα*^-/- ^mice that display a remarkable increase in the CD8^+ ^memory T cells and it was shown that this autoimmune disease is, in contrast to colitis, more dependent on the expression of CD8^+ ^T cells [32,33]. However, this conclusion was derived from study of *CD4*^-/-^*Il2Rα*^-/- ^and CD8^-/-^*Il2Rα*^-/- ^mice without considering the possibility that these mice still have class-II and class-I MHC Ag-restricted T cells independent of the co-receptor expression [33].

### Pancreatitis

Although often associated with IPEX patients [9], Type-1 diabetes was neither observed in Sf mice nor in *Rag1*^-/- ^recipients of Sf lymph node cells. Sf, Sf.*Fas*^*lpr*/*lpr*^, Sf.*Il2*^-/- ^or adult *Rag1*^-/- ^mice that were intravenously transferred with Sf lymph node cells developed very mild pancreatitis with peri-vascular infiltration of leukocytes and occasional destruction of acini outside the islets. Interestingly, moderate to severe pancreatitis with strong leukocyte infiltration and severe destruction of acini was observed when Sf lymph node cells were transferred intraperitoneally into neonatal or adult *Rag1*^-/- ^recipients [25]. Introducing *Sf *gene into NOD mice or BDC2.5 TCR Tg NOD (BDC2.5 is a diabetogenic TCR) facilitated diabetes and therefore the Sf pancreatitis may be one of the contributing factors that accelerate diabetes [24,34]. Using TCR transgenic SF.OT-II (OVA_323–339_-specific, I-A^b^-restricted) mice, we showed that in the absence of Treg, antigen-reactive T cells still require the presence of appropriate antigens with a reasonable binding activity in order to expand, i.e., OT-II clonotypic T cells were not expanded due to the absence of OVA whereas non-clonotypic T cells were expanded by the host antigens [35]. The mouse study suggests that Treg-deficiency plays more of a facilitating role in the disease process so as to impinge diabetes development. As Type-1 diabetes is often observed in IPEX patients, this study suggests that the IPEX patients must have naturally occurring auto-reactive T cells with sufficient binding activity against the islet components present in their system.

### Colitis

Sf mice do not develop colitis and this is most likely due to the weaning condition because Sf.*Fas*^*lpr*/*lpr *^mice that lived beyond weaning develop colitis and transfer of Sf lymph node cells into adult *Rag1*^-/- ^recipients also induced colitis. Transfer into neonate *Rag1*^-/- ^recipient induced little inflammation in the colon before weaning but severe colitis rapidly developed within a few days after weaning [25]. *Il2*^-/- ^mice develop colitis but only after weaning. It is likely that the microbiota present in the colon contributes to the colitis development. Colitis seems to be the easiest organ inflammation induced by adoptive transfer of effector T cells. Even T cells expressing a single transduced TCR often induce colitis and this system has been used to address the "auto-reactive" repertoire of Treg [36]. It is not clear why such T cells often selectively induce colitis but not inflammation in other organs, had they had specificity against a self antigen. The possibility that these TCR recognize food antigens and colon microbiota has been raised [37]. Interestingly, GFP-tagged T cells derived from mice with a *Foxp3 *allele that directs expression of a nonfunctional fusion protein of Foxp3-GFP failed to induce colitis upon transfer into lymphopenic recipients [38].

Integrin α_E_(CD103)β7 is a critical homing and retention receptor for lymphocytes to travel to and lodged in E-cadherin-expressing organs, but CD103^-/-^CD45RB^high ^cells have been shown to induce colitis upon transfer [39]. We generated Sf.*CD103*^-/- ^mice and they have a prolonged lifespan of 7–8 weeks old. They develop colitis and their LN cells were able to transfer colitis to *Rag1*^-/- ^recipients, demonstrating the presence CD103-independent mechanism for colitis [40].

### Myositis

Sf mice do not develop inflammation in the skeletal muscle but direct injection of Sf lymph node cells into the limb muscle of *Rag1*^-/- ^mice induces inflammation not only in the injected sites but also in the skeletal muscle of the other limbs in addition to the organ inflammation normally induced by intravenous transfer [25]. In contrast, intravenous transfer of Sf lymph node cells failed to induce skeletal muscle inflammation. It is possible that directly injected T cells first induced damage of muscle cells and the released antigens were recognized by the specific T cells present in the Sf lymph node cells and expanded. How these T cells travel through the circulation to induce inflammation in other limb muscle remains to be established. Co-transfer of CD25^+ ^Treg with Sf lymph node cells into limb muscle site inhibited myositis and multi-organ inflammation (our unpublished observation). It will be of interest to determine whether these myositis-inducing T cells recognize any of a number of self antigens that have been implicated in myositis in patients [41].

### Inflammation in the accessory reproductive organs

Although Sf.*Fas*^*lpr*/*lpr *^mice have a prolonged lifespan beyond the adult age, they remain reproductive incompetent with low body weight and under-developed reproductive organs. Gross examination revealed a tremendous atrophy in the various accessory reproductive organs. These included coagulation glands/seminal vesicle, preputial glands, epididymis and prostate. Histological examination confirmed the atrophy of these organs, displayed as shrunken glands and empty lumens. This was accompanied with a prominent presence of leukocytic infiltrates in the peri-glandular regions, in the interstitial regions of the testis, and in the regions containing the interstitial cells of the Leydig between the seminiferous tubules. Testosterone treatment successfully restored the growth of the accessory reproductive organs of Sf.*Fas*^*lpr*/*lpr *^mice but the leukocytic infiltrates in the reproductive organs were still present [30].

### Sf mice are useful to study organ inflammation regulation post Treg checkpoint

As Sf mice are totally devoid of Treg, they are ideal to study immune regulation of organ inflammation process independent from the generation of Treg, i.e., at stages after Treg checkpoint. This approach has been used to study how Treg defect impinges on autoimmune disease in NOD mice, inflammatory arthritis in K/BxN mice, and *Aire*^-/- ^mice. In all cases, an accelerated model of autoimmune disease developed, indicating not only a critical control of Treg in autoimmune-prone condition, but also a significant presence of Treg control is present in NOD, K/BxN, and *Aire*^-/- ^mice [24,34,42]. The study of NOD is particularly meaningful as IPEX patients often display type-1 diabetes that is not observed in Sf mice.

Because Treg maintenance requires a normal immune status, a number of immuno-regulators such as *CD28*^-/-^, *Il2*^-/-^, and *Fas*^*lpr*/*lpr *^mutant genes have been bred into Sf mice to study its effect on the multi-organ inflammation response. Introducing *CD28*^-/- ^inhibited Sf inflammation and the hyper-production of IL-4 and IL-10 with prolonged lifespan, suggesting CD28 is important for the response of Tconv in Sf mice [43]. However, the effect on multi-organ inflammation was not addressed in detail and adoptive transfer experiments were not conducted. CD28 has long been implicated as critical regulator for IL-2 production, perhaps in the absence of Treg this requirement becomes less obvious. We introduced *Il2*^-/- ^gene into Sf mice and observed prolongation of lifespan as well. We also observed reduction of IL-4 but more interestingly we observed an increase in CD103 expression on the CD4^+ ^T cells of Sf mice and this increase was blocked in Sf.*Il2*^-/- ^mice, suggesting a requirement of *Il2*^-/- ^on CD103 expression on CD4^+ ^inflammation-inducing T cells in Sf mice [26,40]. Importantly, *Il2*^-/- ^and Sf.*Il2*^-/- ^mice do not display skin and lung inflammation associated with Sf mice, raising the possibility that CD103 controlled by IL-2 is important for skin and lung inflammation in Sf mice [26]. In this respect, Sf mice are ideal to study various factors that are known regulators of organ-specific inflammation not only limited in the areas that control activation of Tconv cells and their effector functions but also the entire inflammation process including T cell trafficking, homing, and retention in the target organs.

Fas (CD95)/FasL (CD178) signaling has been implicated in Treg effector function, reversed T cell signaling for expansion, and apoptosis. We Bred *Il2*^-/- ^and *Fas*^*lpr*/*lpr *^into Sf mice to address these issues and to determine their relative contribution to the spontaneous inflammation process. IL-2 have been shown to be a critical factor for FasL induction on T cells for AICD (activation-induced T cell death) and *Il2*^-/- ^T cells display low AICD compared with normal control in vitro, presumably due to limited activation under the *in vitro *condition [44,45]. However, T cell expression level in Sf.*Il2*^-/- ^and their FasL expression are higher than Sf mice, indicating the *Il2*^-/- ^effect on Sf mice in terms of T cell number and inhibition of inflammation in skin, lung and gastrointestinal organs is not mediated through AICD defect or reverse signaling [46]. In addition, Sf.*Fas*^*lpr*/*lpr *^mice display skin and lung inflammation and develop colitis after weaning but still have a lifespan significantly longer than Sf mice. These observations indicate that the primary reason that Fas/FasL interaction prolongs the lifespan of Sf mice is by inhibiting target organ death in the inflamed tissues.

A conundrum we often asked is why do *Il2*^-/- ^mice have a pattern of multi-organ inflammation different from that of Sf mice, i.e., no inflammation in the ear, skin, lung, and tail [26]. Because *Il2*^-/- ^mice have residual Treg, this phenotype could be caused by difference in organ-specific Treg that regulate the inflammation in these organs. Some studies do support the preferential presence of antigen-specific Treg in the organs and their draining lymph nodes to be responsible for the maintenance of organ-specific tolerance [47,48]. In addition, specific cytokine may regulate inflammation in an organ-specific manner as it has been shown that Treg lacking the ability to produce IL-10 failed to inhibit skin inflammation and colitis [31]. However, these organs remained non-inflamed in Sf.*Il2*^-/- ^mice that totally lacked Treg [26]. This resistance maintained throughout the extended lifespan of Sf.*Il2*^-/- ^mice, indicating a control mechanism operating beyond or independent from the Treg checkpoint [26].

### Scurfy mice are useful to study Treg-controlled inflammation-inducing T cell repertoire

As discussed above, Sf mice contain inflammation-inducing T cells against more than a dozen of organs and potentially other such cells whose organ inflammation-inducing power is limited by TCR-independent mechanisms. Importantly, the large repertoire of inflammation-inducing T cells could be suppressed by the polyclonal Treg, suggesting the presence of an equally large repertoire of Treg TCR [25].

Sf mice CD4^+ ^T cell repertoire as determined by Vβ subgroup distribution failed to detect major differences as compared with B6 control [49], suggesting Sf mice of B6 background do not randomly expand T cells based on Vβ expression and this is consistent with the notion that Treg control polyclonal activation of Tconv cells. However, within each of the Vβ family of Sf mice, marked change in TCR spectratype was observed [49]. This change is pervasive and stochastic. Similar results were observed with *Il2*^-/- ^and *Il2rα*^-/- ^mice and for TCR Vα family members [49]. By using foreign antigen-specific, MHC-II-restricted, TCR Tg mice (OT-II versus Sf.OT-II), the antigen requirement for the expansion of CD4^+ ^T cells in Sf mice was determined [35]. A rapid and selective expansion of the non-clonotypic T cells was observed in Sf.OT-II mice. Expanded populations express various Vβ and Vα family members of the non-Tg origin including T cells lacking the Tg TCR and T cells expressing dual TCR with moderate expression level of the Tg TCR. As clonotypic OT-II Tg T cells are positively selected, the lack of their expansion indicates that the positive selecting antigens are not sufficient to induce expansion even in the absence of Treg. This, in turn, indicates that the antigens responsible for the Tconv expansion in Sf.OT-II mice must be host antigens that include both self antigens as well as foreign antigens that are steadily present in the host such as those present in the air, foods, and microbiota but not rare antigen such as ovalbumin even though the mice contain an extremely high number of ovalbumin-specific Tg T cells. To what extent are the self antigens and foreign antigens contributing to the inflammation in individual organs in Sf mice and their *Rag1*^-/- ^recipients of transfer remains unknown. With such a large repertoire, the identification of the TCR specific targets associated with individual organs may not be easily addressed for Sf mice.

A different case was reported in the Sf mice of Balb/c background [50]. Balb/c mice contain MMTV-encoded viral super-antigens that are recognized by T cells expressing Vβ3, 5, 11, and 12. These T cells (except Vβ11) are deleted to a great extent by negative selection in the thymus although the participation of these Vβ family members in the Treg population is high. However, more than 10-fold increase in Vβ3 or Vβ5 T cells and a significant increase for Vβ11 and Vβ12 T cells are found in the periphery of Balb/c Sf but not normal Balb/c mice, thus providing direct evidence that self antigens can modify Vβ family distribution pattern presumably by inducing proliferation and accumulation of auto-reactive T cells and/or due to lack of Treg-mediated, FasL-based killing of these cells [50]. It will be interesting to determine how big is the repertoire of these T cells and whether they affect the multi-organ inflammation in an organ-specific manner in which the organ strongly produces the MMTV antigens.

## Concluding remark

By breeding and adoptive transfer experiments, Sf mice are shown to contain a large repertoire of inflammation-inducing T cells capable of inducing inflammation in a large number of organs and tissues. Polyclonal Treg can suppress the inflammation when tested, suggesting the presence of an equally large repertoire of functional Treg. An important issue is to determine whether inflammation in individual organs is organ-specific. If so, what are the organ-specific antigens? In this regard, Sf mice should be useful to identify organ-specific Treg either by transferring potential organ-specific Treg into Sf neonates or by co-transfer with Sf lymph node cells into *Rag1*^-/- ^recipients. Sf mice are also useful to study the inflammation process important to the inflammation of various organs. For example, breeding specific an integrin or a chemokine receptor mutant gene into Sf mice could address the importance of the specific gene in the inflammation in various organs at the same time.

## Competing interests

The authors declare that they have no competing interests.

## Authors' contributions

RS did the breeding, histology and transfer experiments. SJS is a long-time collaborator and has participated in the design, implementation, and discussion of the experiments described in this review. SMF and STJ are co-senior authors responsible for the design of experiments and writing of the manuscript.
